# Comparison of antibacterial activity and phenolic constituents of bark, lignum, leaves and fruit of *Rhus verniciflua*

**DOI:** 10.1371/journal.pone.0200257

**Published:** 2018-07-25

**Authors:** Jae Young Jang, Hyeji Shin, Jae-Woong Lim, Jong Hoon Ahn, Yang Hee Jo, Ki Yong Lee, Bang Yeon Hwang, Sung-Ju Jung, So Young Kang, Mi Kyeong Lee

**Affiliations:** 1 College of Pharmacy, Chungbuk National University, Cheongju, Republic of Korea; 2 College of Pharmacy, Korea University, Sejong, Republic of Korea; 3 Department of Aqualife Medicine, Chonnam National University, Yeosu, Republic of Korea; Tallinn University of Technology, ESTONIA

## Abstract

*Rhus verniciflua* is commonly known as a lacquer tree in Korea. The bark of *R*. *verniciflua* has been used as an immunostimulator in traditional medicine, but also causes allergic dermatitis due to urushiol derivatives. For the development of active natural resources with less toxicity, the antibacterial activity of various parts of *R*. *verniciflua* such as bark, lignum, leaves and fruit, together with chemical composition, were investigated. Among the various parts of *R*. *verniciflua*, lignum showed the most potent antibacterial activity against fish pathogenic bacteria such as *Edwardsiella tarda*, *Vibrio anguillarum* and *Streptococcus iniae*. Measurement of total phenolic content and flavonoid content clearly showed a high content of phenolic and flavonoids in lignum among the various parts of *R*. *verniciflua*. Further analysis showed a close correlation between antibacterial activity and phenolic content. In addition, methyl gallate and fustin, the major constituents of bark and lignum, showed antibacterial activity, which suggested phenolic constituents as active constituents. The content of urushiols, however, was highest in bark, but there was a trace amount in lignum. LC-MS-MS and PCA analysis showed good discrimination with the difference of phenolic composition in various parts of *R*. *verniciflua*. Taken together, phenolic compounds are responsible for the antibacterial activity of *R*. *verniciflua*. The lignum of *R*. *verniciflua* contains high content of phenolic compounds with less urushiols, which suggests efficient antibacterial activity with less toxicity. Therefore, the lignum of *R*. *verniciflua* is suggested as a good source for antibacterial material to use against fish bacterial diseases.

## Introduction

Fish is rich in nutrients including protein, vitamins, minerals and polyunsaturated fatty acids. Due to the high consumption of fish, aquaculture is a major global industry that has developed rapidly in a short period of time. However, industrial aquaculture is susceptible to diverse infections caused by overcrowded rearing and excessive feeding for mass production within an intensive aquaculture system. The infection of fish with bacteria or viruses results in serious economic losses due to its high mortality. Moreover, increased outbreaks of disease require more use of antibiotics and chemicals, which gives rise to resistance to antibiotics and food safety issues. Therefore, the demand for antibiotics with strong potency and less toxicity has risen sharply, and natural products have been suggested as alternatives to chemical antibiotics [1–3]. Natural products are considered to be safe for both fish and humans and exert little resistance to bacteria [4]. In addition, natural products contain various constituents with different skeletons such as flavonoids, terpenoids, xanthones, alkaloids and polysaccharides. The diversity of structures enables a wide range of pharmacological effects including antioxidant, anticancer, anti-inflammatory and neuroprotective activity, and also contributes synergic activities [5–6]. Recently, several natural substances such as bee venom, essential oil and phenolic compounds have been reported to have antimicrobial agents [7–9].

**Fig 1:**
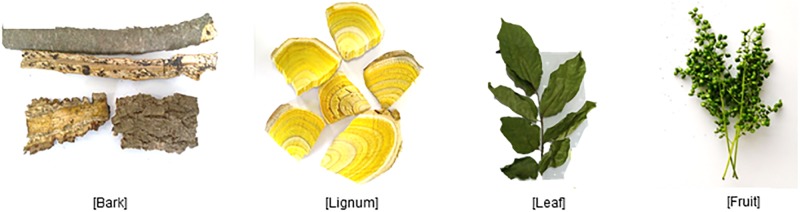
Representative photographs of bark, lignum, leaves and fruit of *Rhus verniciflua*.

*Rhus verniciflua* Stokes (Anacardiaceae) is a plant native to East Asian countries, including Korea. It is also known as a lacquer tree and is used in traditional herbal medicine. The bark of this tree has been used as an immunostimulant in folk medicine and various biological activities including antioxidant, anticancer, anti-inflammatory and antimicrobial effects have been reported [10–13]. In our previous study, extracts and fractions of the bark of *R*. *verniciflua* showed significant antibacterial activity against fish pathogen bacteria such as *Edwardsiella tarda* and *Vibrio anguillarum*. The bark of *R*. *verniciflua* and its flavonoids also have antiviral activities against fish pathogenic viruses [14–15]. Therefore, *R*. *verniciflua* is suggested to have potential as antimicrobial therapeutics against fish infectious diseases. However, the bark of *R*. *verniciflua* is consumed at a high price due to small supply. In addition, its use has been limited due to the presence of allergic components, urushiols, in the bark. Other parts of this plant are also consumed as food ingredients or alcoholic beverages in traditional use; however, few investigations have been carried out regarding the composition and biological activity of various parts of *R*. *verniciflua*. In particular, no studies have been conducted to evaluate the antibacterial activity of each part of *R*. *verniciflua* against fish pathogens. Generally, different parts of plants contain different types of constituents that contribute diverse biological activities. Therefore, we compared the antibacterial activities of various parts of *R*. *verniciflua* such as bark, lignum, leaves and fruit (Fig 1) for development as an alternative to antibiotics for fish pathogens. The effects of phenolic contents and its major compounds on antibacterial activity were also investigated.

## Materials and methods

### Plant materials

The bark, lignum and leaves of *R*. *verniciflua* were collected from four different regions in Korea, such as Wonju, Okcheon, and Buyeo. No specific permissions were required for access because these locations were privately owned and the field studies did not involve endangered or protected species.

The materials were air-dried at room temperature for 2 weeks and pulverized and extracted with MeOH using sonic apparatus for 2 hrs. Voucher specimens were deposited in a specimen room of the herbarium of the College of Pharmacy at Chungbuk National University.

### Measurement of antibacterial activity

#### Bacteria and culture conditions

*Streptococcus iniae* KCTC 3657, *Vibrio anguillarum* KCTC 2711 and *Edwardsiella tarda* KCTC 12267 were purchased from the Korean Collection for Type Cultures (Daejeon, Korea). For antimicrobial susceptibility tests, strains were cultured on Brain Heart Infusion Agar (BHIA) in an incubator at 25 °t for 24 h. Bacterial colonies taken directly from BHIA plates were incubated in Brain Heart Infusion Broth (BHIB) at 25 ° for 24 h. From this culture, a suspension equivalent to a 0.5 McFarland standard in BHIB was prepared.

#### Disc diffusion assay

The extracts of different parts from RVS were tested with a disc diffusion assay [15]. Bacterial suspensions with a turbidity equivalent to 0.5 McFarland standard were prepared as described above. The bacterial inocula of 100 μl (10^8^ CFU/ml) were seeded into BHI agar using a disposable spreader. After drying, filter paper discs (6 mm in diameter) impregnated with the extracts (2 mg/disc) were placed on test bacteria-inocula plates. Commercially available discs (Oxoid Ltd., Basingstoke, Hampshire, UK) containing 30 μg oxytetracycline each were used as a positive control. Plates were incubated at 25 °5 for 48 h and the clear zones including the diameter of the disc (mm) were measured with a digital caliper.

#### Microdilution method—Minimum inhibitory concentration (MIC)

The compounds were serially diluted with BHIB in a 96-well plate. An equal volume of bacterial suspension (1x10^6^ CFU/ml) was added to the wells to give a final volume of 200 μl. The plate was incubated at 25°. for 24 h. Appropriate controls included the solvent used to dissolve the samples, broth alone and the antibiotics amoxicillin and oxytetracycline as positive controls. The lowest concentration of samples that visibly inhibited bacterial growth was considered the minimum inhibitory concentration (MIC). Each assay was repeated three times.

### Determination of total flavonoid content

An aluminum chloride colorimetric assay was employed for the measurement of the total flavonoid content in the samples. Briefly, samples were prepared in a 96-well plate and 5% NaNO_3_ was added to the reaction mixture. The reaction mixture was kept in incubation for 5 min, and 10% AlCl_3_ was added. After incubation with gentle shaking, 1 N NaOH and H_2_O was added to the reaction plate. Absorbance at 510 nm was measured with a microplate reader. The total flavonoid content of each sample was expressed as catechin equivalent (CE) using catechin as a standard.

### Determination of total phenolic content

A Folin-Ciocalteu assay was employed for the determination of the total phenolic content. The reaction was started with the addition of Folin-Ciocalteu’s phenol reagent to the 96-well plate containing the test samples. The reaction mixture was incubated for 5 min with gentle shaking, and then 7% Na_2_CO_3_ was added to the reaction mixture. The reaction mixture was kept in a dark condition at room temperature for reaction. After 90 min of incubation, the absorbance was measured at 630 nm with a microplate reader. The total phenolic content in each sample was expressed as gallic acid equivalent (GAE) using gallic acid as a standard.

### LC-Q-TOF MS/MS analysis

LC-Q-TOF MS/MS analysis was performed on an Agilent 1260 series system (Agilent, Santa Clara, CA, USA) connected to an Agilent 6530 Q-TOF mass spectrometer (Agilent, Santa Clara, CA, USA). The HPLC system was equipped with an auto-sampler, binary pump, degasser, and diode array detector. UV spectra was monitored at 220, 254, and 300 nm. The mass spectrometer was equipped with electrospray ionization (ESI) in the negative mode. The MS and MS/MS spectra were obtained with a mass range of *m/z* 50–1700. The collision energy for MS/MS fragmentation was set at 10, 20, 30, and 40 V. MassHunter Workstation software LC/MS Data Acquisition for 6530 series Q-TOF (version B.05.00) was applied to adjust all the acquisition parameters. The chromatographic separation of the sample was performed on a Shiseido CapCell PAK C18 column (5μm, 4.6 mm I.D. × 150 mm) with a C18 guard column (4.00 × 3.00 mm; Phenomenex, USA). The mobile phase consisted of water containing 0.1% formic acid (solvent A) and acetonitrile containing 0.1% formic acid (solvent B). The gradient elution was 0–5 min, 5% B, 5–30 min, 5–95% B. The injection volume was 5 μl, and the flow rate was 0.6 ml/min. Principal component analysis (PCA) and statistical analysis were performed to evaluate the differences between samples using Mass Profiler Professional (MPP).

### Isolation of the compounds

Compounds **8** and **14**, major compounds from bark and lignum, were isolated as previously reported [15]. The leaves of *R*. *verniciflua* (60 g) were extracted twice with 100% MeOH, which yielded the methanol extract (10.1 g). The methanol extract was suspended in H_2_O and partitioned successively with *n*-hexane, CH_2_Cl_2_, EtOAc, and *n*-BuOH. The EtOAc fraction of the leaves (RVLE, 1.2 g) was subjected to Sephadex LH-20 and eluted with n-hexane:CH_2_Cl_2_:MeOH (5:5:1) to give nine subfractions (RVLE1- RVLE9). The RVLE6 was subjected to medium-pressure liquid chromatography (MPLC) over silica gel and eluted with a mixture of CH_2_Cl_2_-MeOH to give five subfractions (RVLE6A- RVLE6E). Compound **15** (12.0 mg) was obtained from RVLE6D by semi-preparative HPLC eluting with acetonitrile-water (27:73).

## Results and discussion

### Preparation of samples of different parts of *R*. *verniciflua*

Four parts of *R*. *verniciflua*, bark, lignum, leaves and fruit, were collected from different regions of Korea, including Wonju, Okcheon and Boeun (Fig 1). For the comparison of chemical patterns and biological activity, each part was extracted with 80% MeOH, respectively.

### Antibacterial activity against fish pathogens

The antibacterial activity against fish pathogens was determined using the disc diffusion method. *E*. *tarda*, *V*. *anguillarum* and *S*. *iniae* were selected as fish pathogenic bacteria and antibacterial activity was assessed by measuring the diameter of the inhibition zone formed around paper discs containing an extract from each part of *R*. *verniciflua* (2 mg/disc).

As shown in Table 1, the antibacterial activity was differed substantially depending on the plant part and bacteria tested. Among four different parts, the lignum of *R*. *verniciflua* showed the strongest activity against all bacteria tested, with inhibition zone diameters ranging from 10.38 to 11.09 mm. Fruit and bark showed similar antibacterial potency against *V*. *anguillarum* and *S*. *iniae*, with inhibition zone diameters ranging from 8.62 to 8.93 mm; however, only bark showed antibacterial activity against *E*. *tarda*. Leaves showed only weak antibacterial activity against *V*. *anguillarum* and *S*. *iniae*.

**Table 1:** Antibacterial activity of bark, lignum, leaves and fruit of *R*. *verniciflua* against *E*. *tarda*, *V*. *anguillarum* and *S*. *iniae*.

Samples (2 mg/disc)	Clear zone (mm)		
	*E*. *tarda*	*V*. *anguillarum*	*S*. *iniae*
Bark	7.39 ± 0.10	8.64 ± 1.36	8.93 ± 1.15
Lignum	10.38 ± 0.70	10.50 ± 0.46	11.09 ± 0.56
Leaf	No Effect	6.78 ± 0.26	7.68 ± 0.72
Fruit	No Effect	8.62 ± 0.82	8.50 ± 1.10
Positive control (OTC) ^a)^	20.03 ± 0.68	20.23 ± 0.50	19.64 ± 0.42

^a)^ Oxytetracycline was used as the positive control

### Total phenolic and flavonoid contents

**Fig 2:**
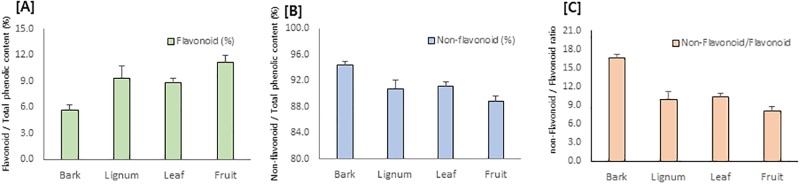
Ratio of flavonoid, non-flavonoid and non-flavonoid/flavonoid of extracts of bark, lignum, leaves and fruit of *R*. *verniciflua*.

*R*. *verniciflua* has been reported to contain various polyphenols, including flavonoids and phenolic compounds, and they contribute to its diverse biological activity [16–19]. Therefore, the total phenolic content of each part was analyzed. Consistent with previous studies [10–12], *R*. *verniciflua* is rich in phenolic compounds. The amount of total phenolic compounds, however, differs depending on the plant part and ranges from 68.9 to 363.6 mg GAE/g extract. Among the parts of *R*. *verniciflua*, lignum contains the highest amounts of phenolic constituents, followed by bark, leaves and fruit (Table 2). Lignum extract contains up to 363.6 mg GAE/g, which is more than twice that of leaves and bark. Total flavonoid content and non-flavonoid content also showed a similar pattern, which is the most abundant in lignum. Interestingly, however, the ratio of non-flavonoid and flavonoid showed a differential pattern. Bark contains relatively high non-flavonoid content, with a non-flavonoid/flavonoid ratio of 16.4, whereas other parts contain relatively high flavonoid content with a non-flavonoid/flavonoid ratio of 8.1 to 10.9 (Fig 2). Taken together, not only the total amount of phenolic and flavonoid content, but also the composition of each constituent differs depending on the part of *R*. *verniciflua*.

**Table 2:** Total phenolic, flavonoid and non-flavonoid contents in the extracts of bark, lignum, leaves and fruit of *R*. *verniciflua*.

	Parts of *R*. *verniciflua*			
	Bark	Lignum	Leaf	Fruit
Total phenolic	151.7 ± 38.3	363.6 ± 80.6	127.7 ± 7.7	68.9 ± 7.7
Flavonoid	8.6 ± 1.9	35.0 ± 12.1	11.2 ± 0.2	7.7 ± 1.5
Non-flavonoid	143.1 ± 36.3	328.6 ± 68.6	120.2 ± 12.4	61.1 ± 6.2

### Correlation between biological activity and phenolic content

**Fig 3:**
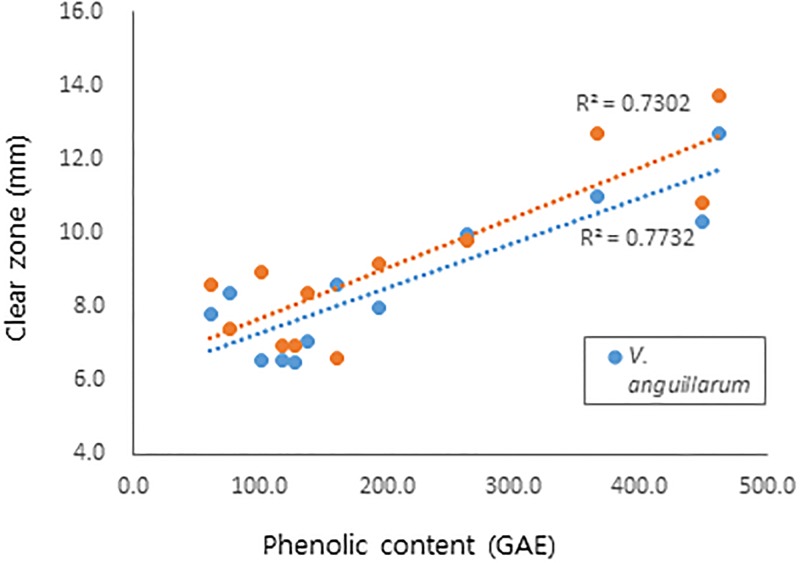
Correlation between antibacterial activity and phenolic contents of *R*. *verniciflua*.

Polyphenols are known to exert diverse biological activities, including antioxidant and antibacterial activities [19–22]. Therefore, the effect of total polyphenol content on antibacterial and antioxidant activity was analyzed. As shown in Fig 3, antibacterial activities against *V*. *anguillarum* and *S*. *iniae* showed a correlation with total phenolic content, with *R*^*2*^ of 0.7732 and 0.7302, respectively. These results suggest phenolic constituents as an active ingredient of the antibacterial activity of *R*. *verniciflua*.

### Chemical profiles of bark, lignum, leaves and fruit

**Fig 4:**
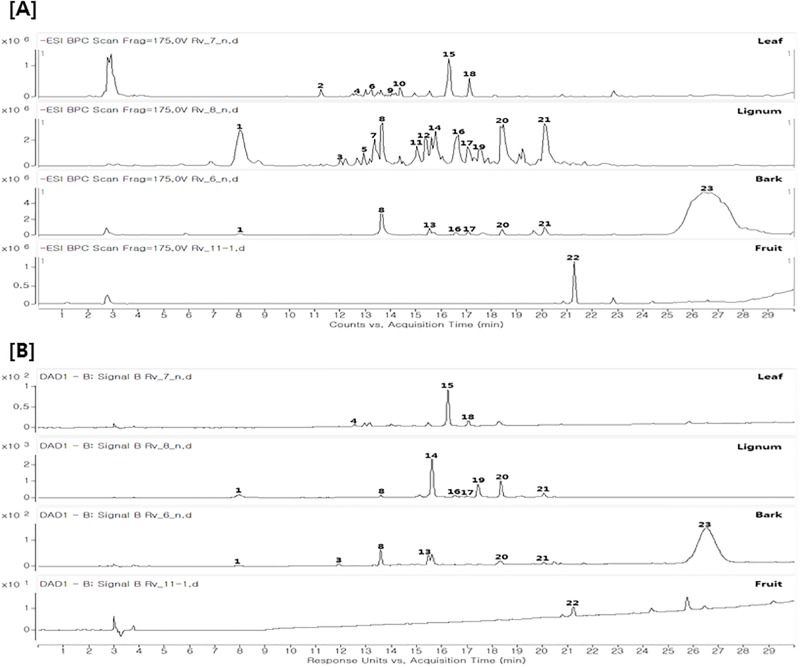
HPLC chromatogram of bark, lignum, leaves and fruit of *R*. *verniciflua*. (A) Mass chromatogram (negative mode), (B) UV chromatogram (254 nm).

The chemical profiles of bark, stem, leaves and fruit of *R*. *verniciflua* were first analyzed using LC-MS/MS analysis and a database (Fig 4). Twenty-three compounds were detected in *R*. *verniciflua* (S1 and S2 Figs) and 13 compounds were tentatively identified based on retention times, UV spectra, and MS data (accurate mass, MS/MS fragments) (Table 3). As expected, phenolic compounds including flavonoids are a major group in *R*. *verniciflua*. However, the chemical profiles of each part were totally different.

**Table 3:** Compounds identified in the extract of bark, lignum, leaves and fruit of *R*. *verniciflua* by LC-MS/MS analysis.

Peak No.	Compounds identification	t_R_ (mins)	observed *m/z*	calculated *m/z*	Molecular formula [M-H]^-^	MS/MS fragments (*m/z*)	UV (λmax, nm)	Detected parts ^a)^
**1**	Gallic acid	8.0320	169.0153	169.0142	C_7_H_5_O_5_	125 [M-CO_2_-H]^-^	271	BK, LG
**2**	Unidentified	11.2310	315.1107	315.1085	C_14_H_19_O_8_	153 [M-C_6_H_10_O_5_-H]^-^		LF
**3**	Dihydroxybenzoic acid	11.9810	153.0202	153.0193	C_7_H_5_O_4_	109 [M-CO_2_-H]^-^	217, 261, 297	B, L
**4**	Unidentified	12.6050	297.0636	297.0616	C_13_H_12_O_8_	135 [M-C_6_H_10_O_5_-H]^-^	291, 325	LF
**5**	Unidentified	12.9230	579.1552	579.1567	C_23_H_31_O_17_	137 [M-442-H]^-^	280	LG
**6**	Unidentified	13.2300	353.0905	353.0878	C_16_H_17_O_9_	191 [M-C_6_H_10_O_5_-H]^-^	294, 325	LF
**7**	Unidentified	13.3590	579.1548	579.1567	C_23_H_31_O_17_	137 [M-442-H]^-^	281	LG
**8**	4-methyl gallate	13.6510	183.0309	183.0299	C_8_H_7_O_5_	124 [M-C_2_H_3_O_2_-H]^-^	275	BK, LG, LF
**9**	Unidentified	14.0420	297.0636	297.0616	C_13_H_12_O_8_	135 [M-C_6_H_10_O_5_-H]^-^	316	LF
**10**	Unidentified	14.4170	337.0957	337.0929	C_16_H_17_O_8_	191 [M-C_6_H_10_O_4_-H]^-^	313	LF
**11**	Unidentified	15.0460	287.0582	287.0561	C_15_H_11_O_6_	109 [M-178-H]^-^		LG
**12**	Unidentified	15.3580	607.1866	607.1880	C_25_H_35_O_17_	271 [M-336-H]^-^	280	LG
**13**	Pentagalloyl glucose	15.5510	939.1188	939.1103	C_41_H_31_O_26_	770 [M-C_7_H_5_O_5_-H]^-^	279	BK, LG
**14**	Fustin	15.7950	287.0585	287.0561	C_15_H_11_O_6_	109 [M-C_9_H_6_O_4_-H]^-^	218, 231, 279	BK, LG
**15**	Quercitrin	16.2910	447.0964	447.0933	C_21_H_19_O_11_	301 [M-C_6_H_10_O_4_-H]^-^	258, 351	LF
**16**	Taxifolin	16.6700	303.0533	303.0510	C_15_H_11_O_7_	285 [M-H_2_O-H]^-^	290	BK, LG
**17**	Garbanzol	17.0440	271.0635	271.0612	C_15_H_11_O_5_	243 [M-CO-H]^-^	213, 276, 311	BK, LG
**18**	Kaempferol-3-*O*-rhamnoside	17.1650	431.1017	431.0984	C_21_H_19_O_10_	285 [M-C_6_H_10_O_4_-H]^-^	262, 344	LF
**19**	Fisetin	17.5440	285.0427	285.0405	C_15_H_9_O_6_	135 [M-C_8_H_6_O_3_-H]^-^	316, 359	BK, LG
**20**	Sulfuretin	18.4810	269.0477	269.0455	C_15_H_9_O_5_	133 [M-C_8_H_8_O_2_-H]^-^	257, 269	BK, LG
**21**	Butein	20.1050	271.0634	271.0612	C_15_H_11_O_5_	135 [M-C_8_H_8_O_2_-H]^-^	261, 380	BK, LG
**22**	Unidentified	21.2620	541.1168	541.1140	C_30_H_21_O_10_	311 [M-230-H]^-^	225	FR
**23**	Urushiol (3-pentadecyl catechol, double bond = 3)	26.7350	313.2198	313.2173	C_21_H_29_O_2_	122 [M-C_14_H_23_-H]^-^	225	BK

^a)^ BK, bark; LG, lignum; LF, leaf; FR, fruit

**Fig 5:**
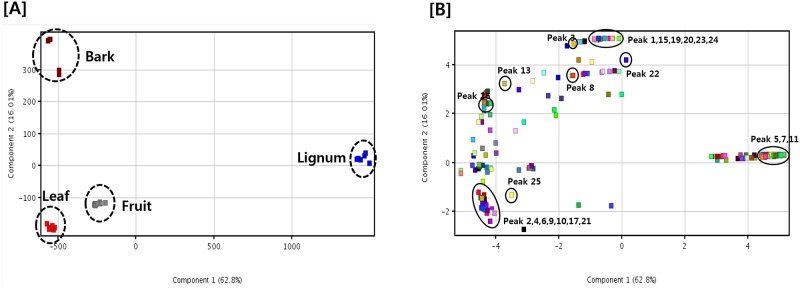
PCA results of the extracts of bark, lignum, leaves and fruit of *R*. *verniciflua*. (A) Score plot and (B) loading plot.

To better analyze and visualize the similarities and differences among each part, multivariate data analyses were employed. Principal component analysis (PCA) was first applied to classify the patterns of each part. PCA is an unsupervised clustering process for identifying patterns by reduction of the number of dimensions. It is widely used for the classification of various samples with respect to designated criteria [23, 24]. As shown in Fig 5, discrimination between each part was well performed. The PCA score plot discriminated lignum from other parts, with separate clustering on the positive score value of PC1, whereas other parts were positioned on the negative side of PC1 (Fig 5). Further analysis using the corresponding loading plot of PC1 suggested the most discriminatory constituents in lignum as peaks 5, 7 and 11, which contained high amounts in lignum compared to other parts. Score plot and corresponding loading plot also showed that leaves was separated from other parts by peaks 2, 4, 6, 9, 10, 17 and 21.

### Identification of major constituents of bark, lignum, leaves and fruit

Comparison of the HPLC chromatogram of *R*. *verniciflua* showed differences depending on the part. HPLC analysis also suggested the presence of major characteristic compounds in each part of *R*. *verniciflua*. For the verification of its major characteristic compounds and evaluation of antibacterial activity, further isolation was conducted using chromatographic techniques. The structures of compounds were identified by spectroscopic analysis including NMR data and UV spectrum, and by the comparison of references [15, 25].

**Fig 6:**
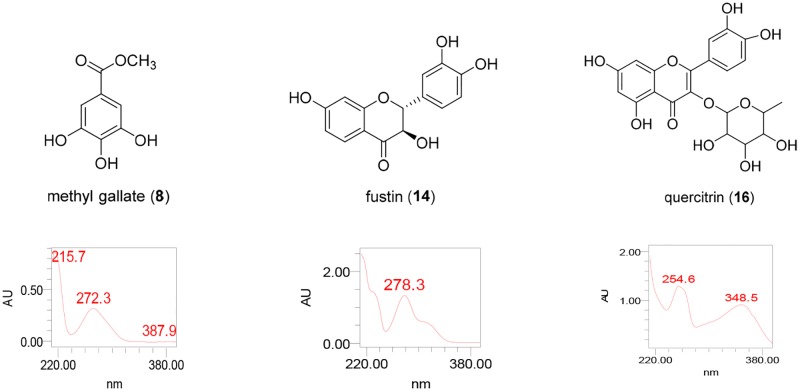
Chemical structures of methyl gallate (8), fustin (14) and quercitrin (15), major constituents of bark, lignum and leaves, respectively.

The major constituents of bark, lignum and leaves were identified as methyl gallate (**8**), fustin (**14**) and quercitrin (**15**) (Fig 6), respectively, which was consistent with LS-MS/MS analysis. These three major constituents are phenolic compounds, but they can be divided into further subtypes. Fustin (**14**) and quercitrin (**15**) are flavonoids, whereas methyl gallate (**8**) is a simple phenolic compound. These results are consistent with Fig 3C, which supported the differential composition of phenolic content in each part of *R*. *verniciflua*. The major constituent of fruit, however, could not be identified in our present study.

### Evaluation of antibacterial of major constituents of bark, stem and leaves

Next, the antibacterial activity of the major constituents of bark, lignum and leaves was tested (Table 4). All three major constituents are phenolic compounds, but the activities were quite different. Methyl gallate (**8**) showed the most potent antibacterial activity. Fustin (**14**) also showed mild antibacterial activity against *E*. *tarda*; however, quercitrin (**15**) showed only weak activity against *V*. *anguillarum* and *S*. *iniae*. Considering the biological activity of the extract and major constituents of each part of *R*. *verniciflua*, they showed different patterns. The lignum of *R*. *verniciflua* was the most active among the parts, but methyl gallate (**8**), the major constituent of bark, was more potent than fustin (**14**), and that of lignum. Although the antibacterial activity of the major constituent of lignum is less potent than that of bark, the phenolic content in lignum is much higher than in bark. From these results, we suppose that a high content of phenolic compounds with moderate activity might contribute to the potent antibacterial activity of lignum compared to other parts.

**Table 4:** Antibacterial activity of major constituents of bark, lignum and leaves of *R*. *verniciflua* against *E*. *tarda*, *V*. *anguillarum* and *S*. *iniae*.

Compound	Antibacterial activity (MIC, μg/ml)		
	*E*. *tarda*	*V*. *anguillarum*	*S*. *iniae*
Methyl gallate (**8**)	31.25	2000	> 2000
Fustin (**14**)	1000	1000	1000
Quercitrin (**15**)	> 2000	2000	2000
Positive control (OTC) ^a)^	0.5	0.25	0.25

^a)^ Oxytetracycline was used as the positive control

### Comparison of biological activity and chemical constituents of bark, stem, leaves and fruit

A phytochemical investigation of four different parts of *R*. *verniciflua* revealed that phenolic constituents are major compounds of *R*. *verniciflua*. The amount of total phenolic compounds was highest in lignum, followed by bark, leaves and fruit. Interestingly, the bark contains a relatively high portion of non-flavonoids compared to other parts, as derived from a high ratio of non-flavonoid/flavonoid (Fig 3). The chemical profiles of bark, stem, leaves and fruit of *R*. *verniciflua* analyzed using LC-MS/MS and database analysis consistently suggested phenolic compounds as major constituents of this plant. Further purification yielded major compounds such as methyl gallate (**8**) from bark, fustin (**14**) from lignum and quercitrin (**15**) from leaves. All three major constituents belong to phenolic compounds, and further can be divided into methyl gallate (**8**) as a simple phenolic compound and fustin (**14**) and quercitrin (**15**) as flavonoids, which is consistent with our analysis.

Concerning antibacterial activity, lignum showed the most potent activity, followed by bark, leaves and fruit, which is the same order as phenolic contents. Further analysis revealed a positive correlation between antibacterial activity and phenolic content of each part of *R*. *verniciflua*. Among the major constituents, methyl gallate (**8**) from bark was the most effective, followed by fustin (**14**) from lignum. Therefore, phenolic compounds are responsible for the biological activity of each part of *R*. *verniciflua*; however, the type of constituent and potency of its biological activity are quite different depending on the part. In addition, fruit has less phenolic compound contents compared to bark and leaves, but showed similar or better activity. Previous studies reported that glycoproteins are active constituents of the fruit of *R*. *verniciflua* [26, 27]. In our present study, few compounds were detected in our LC-MS-MS analysis of the fruit of *R*. *verniciflua*, and their structures could not be identified. Taken together, the presence of other types of constituents in the fruit of *R*. *verniciflua* were suggested, which needs to be clarified by further study.

The similarities and differences between each part of *R*. *verniciflua* were also analyzed by PCA in the present study. The PCA score plot showed good discrimination between parts. Lignum was discriminated from other parts with the positive score values of PC1 and peaks 5, 7, and 11 are suggested as discriminant compounds. Bark also formed a cluster with the positive score values of PC2. Moreover, urshiol, an allergic component, is one of the discriminant constituents in bark, and is contained in high amounts in bark compared to lignum, leaves and fruit. These results suggested that, contrary to bark, other parts can be developed for functional products with fewer side effects.

Comparative analysis of different parts of plants showed differential composition and biological activity depending on the part [28, 29]. In the case of *Salvia miltiorrhiza*, tanshinones and phenolic acids are abundant in roots, whereas flavonoids and triterpenes are abundant in stems and leaves. The composition of flowers of *S*. *milriorrhiza* was quite dependent on growth stage. Different parts of plants and plant waste materials have been developed as alternatives for drug development with better efficacy and economic advantages. However, information about the constituents and biological activity of different plant resources is quite limited. Our present study clearly showed the differences in chemical composition and antibacterial activity between each part of *R*. *verniciflua*, which was supported by PCA analysis. We further characterized the major constituents of bark, lignum, leaves of *R*. *verniciflua* and their antibacterial activity. Therefore, our present study provided a basis for the use of other parts of *R*. *verniciflua*. In particular, lignum showed strong antibacterial activity against *E*. *tarda*, *V*. *anguillarum* and *S*. *iniae* and high phenolic contents and less urshiol compared to bark, which can increase biological activity and reduce allergic toxicity. Therefore, we carefully suggest that the lignum of *R*. *verniciflua* can be a good candidate for the development of antibacterial agents against fish diseases.

## Conclusions

We investigated the chemical composition and antibacterial activity of different parts of *R*. *verniciflua*, which were bark, lignum, leaves and fruit. Among them, lignum showed strong antibacterial activity against *E*. *tarda*, *V*. *anguillarum* and *S*. *iniae* and phenolic contents were suggested as active constituents. The similarities and differences among each part were analyzed by PCA and good discrimination between each part was observed in the PCA score plot. Lignum formed a uniform cluster in the PCA score plot and the loading plot showed that lignum contains little content of urshiol, an allergic component of this plant. Therefore, the lignum of *R*. *verniciflua* can be a good candidate for the development of antibacterial agents against fish pathogens.

## Supporting information

S1 Fig MS and UV (220, 254, 300 nm) chromatograms of *R*. *verniciflua* extract. (PPTX)

S2 Fig MS and UV spectrum of compounds identified in *R*. *verniciflua* extract by LC-MS/MS analysis. (PPTX)
