# Supplementary material for: Comparison of antibacterial activity and phenolic constituents of bark, lignum, leaves and fruit of Rhus verniciflua
Source: PLoS One. 2018 Jul 25;13(7):e0200257. doi: 10.1371/journal.pone.0200257 (PMC6059415; doi:10.1371/journal.pone.0200257)
Supplement: S1 Fig — (PPTX) [file pone.0200257.s001.pptx]

## Slide 1
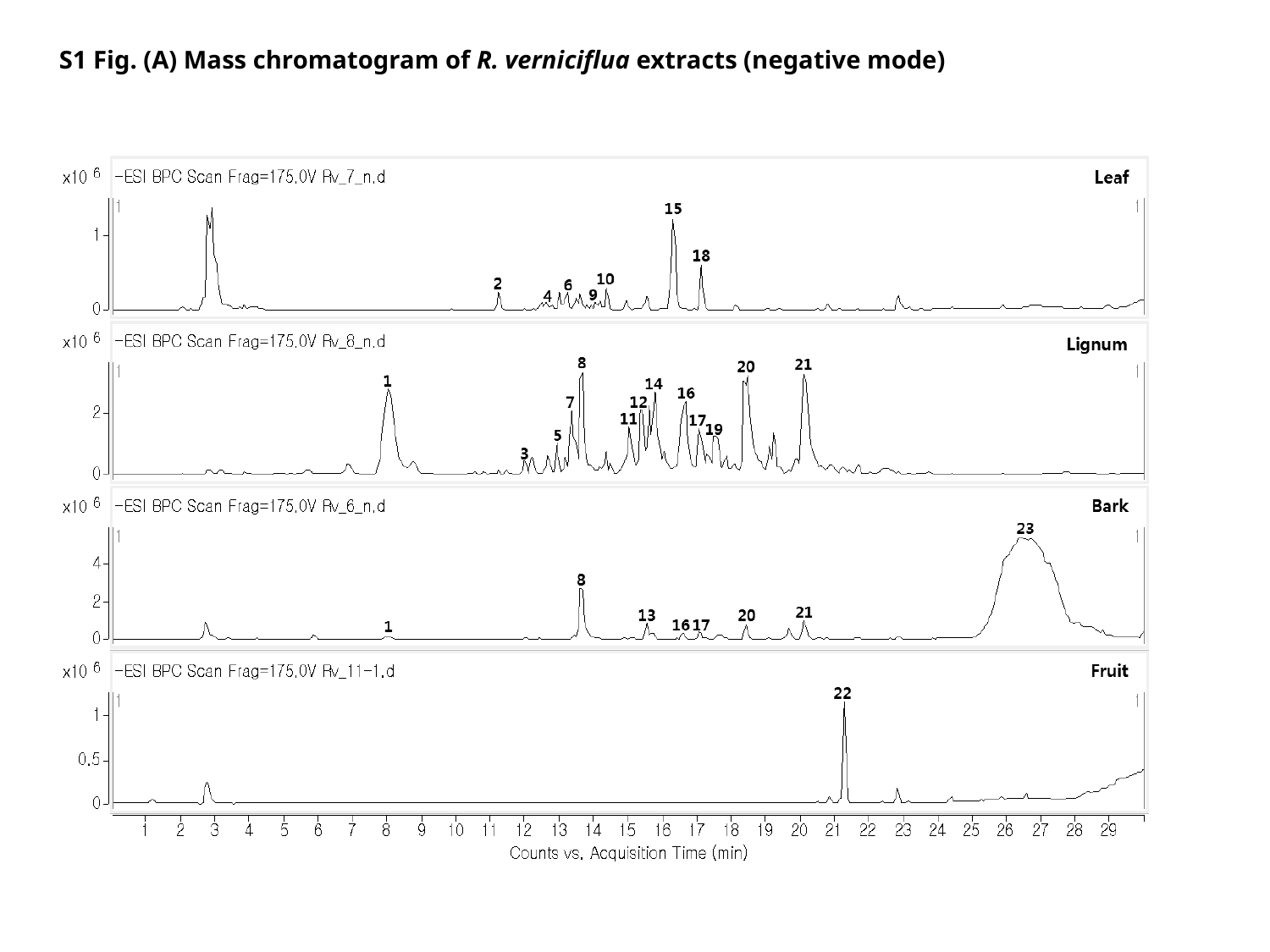

S1 Fig. (A) Mass chromatogram of R. verniciflua extracts (negative mode)

## Slide 2
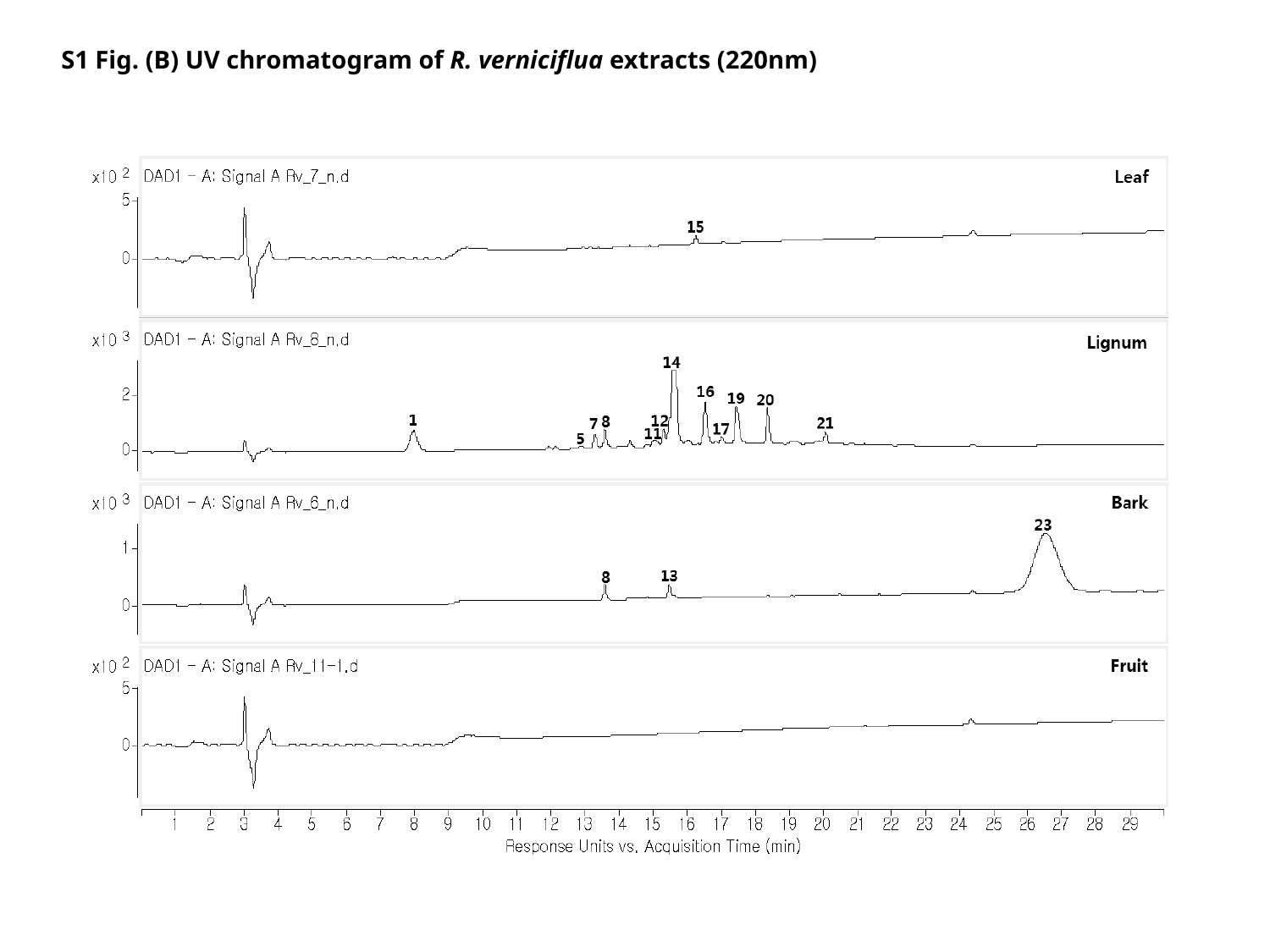

S1 Fig. (B) UV chromatogram of R. verniciflua extracts (220nm)

## Slide 3
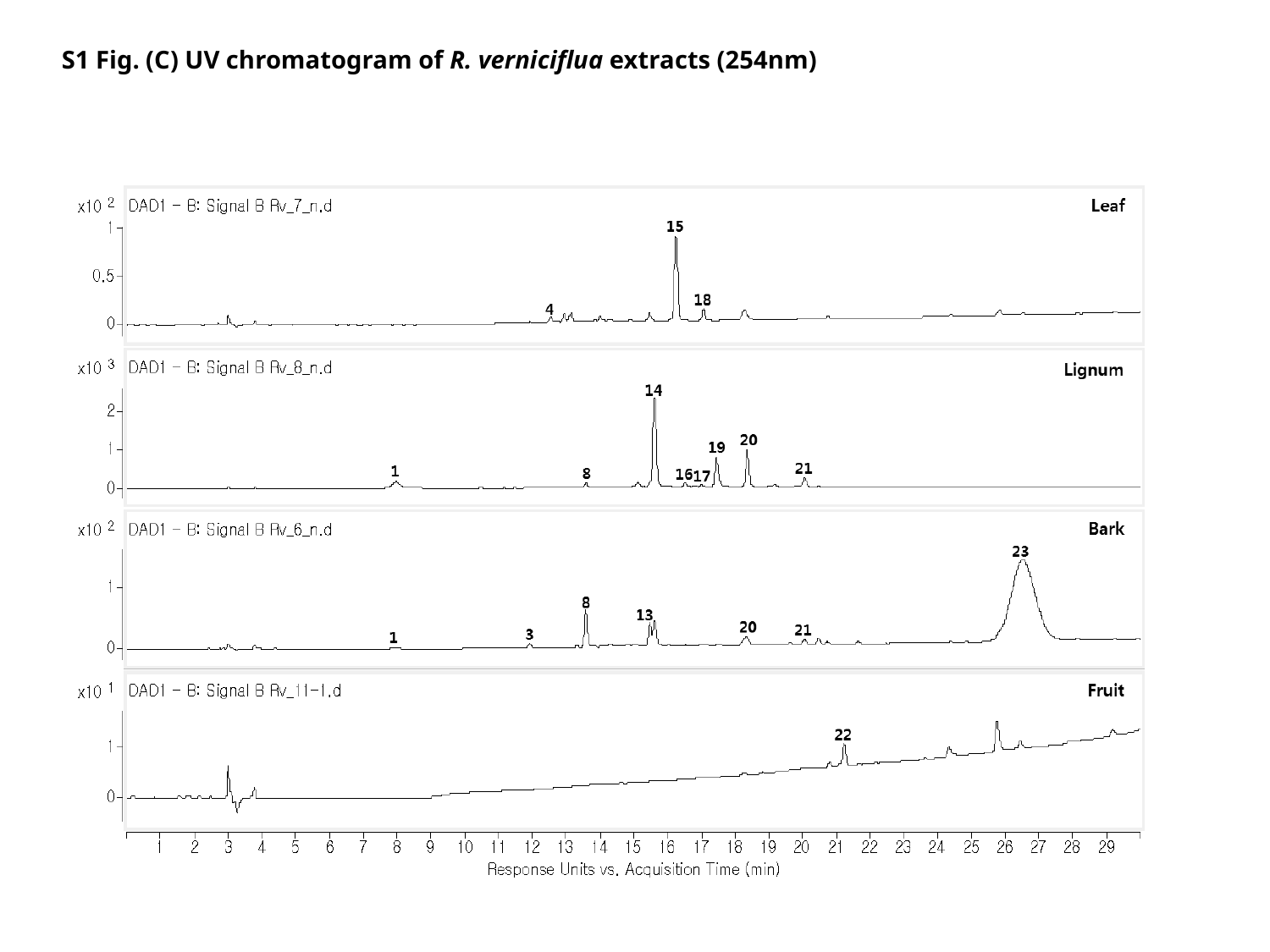

S1 Fig. (C) UV chromatogram of R. verniciflua extracts (254nm)

## Slide 4
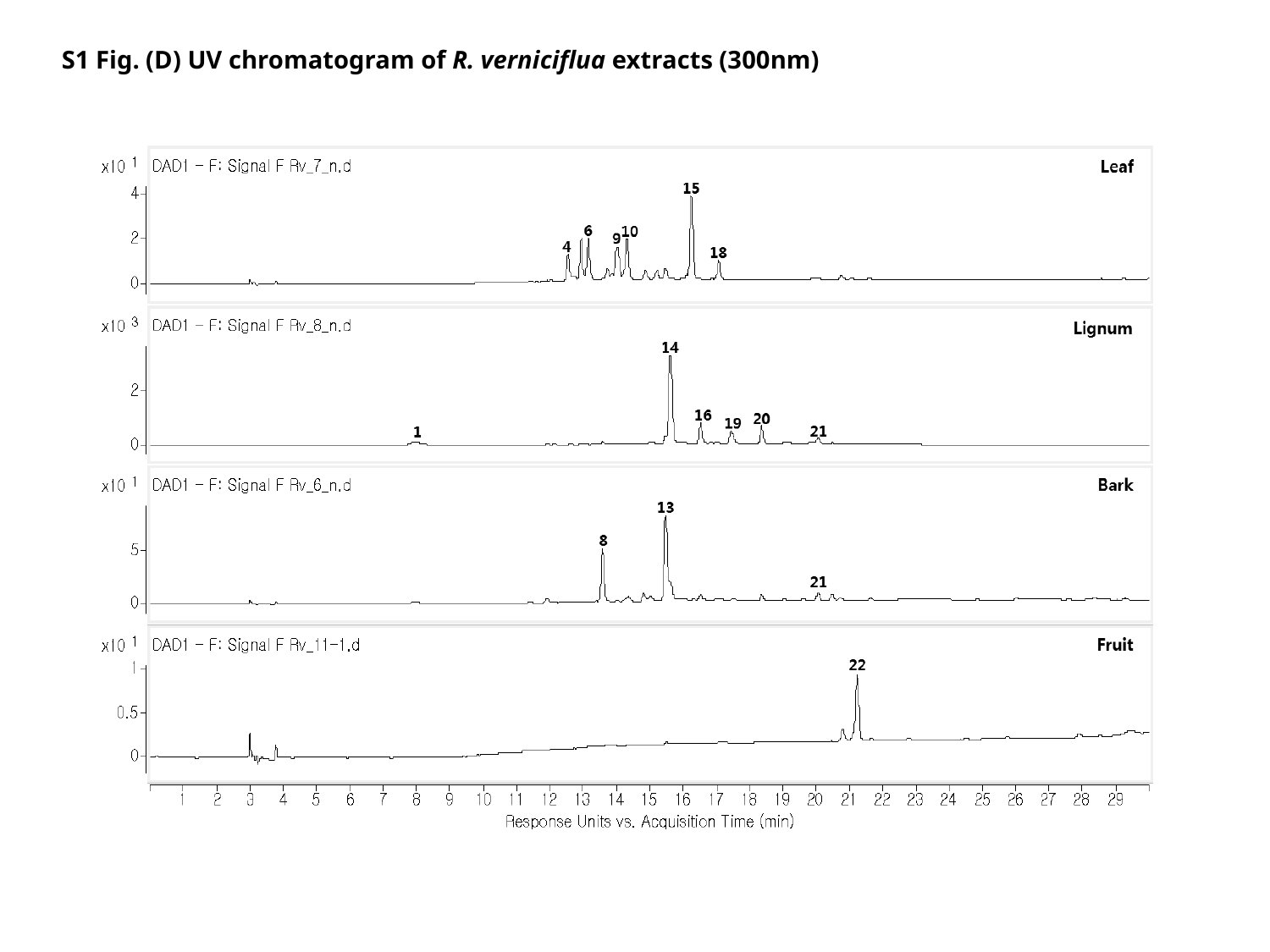

S1 Fig. (D) UV chromatogram of R. verniciflua extracts (300nm)
