# Supplementary material for: Comparison of antibacterial activity and phenolic constituents of bark, lignum, leaves and fruit of Rhus verniciflua
Source: PLoS One. 2018 Jul 25;13(7):e0200257. doi: 10.1371/journal.pone.0200257 (PMC6059415; doi:10.1371/journal.pone.0200257)
Supplement: S2 Fig — (PPTX) [file pone.0200257.s002.pptx]

## Slide 1
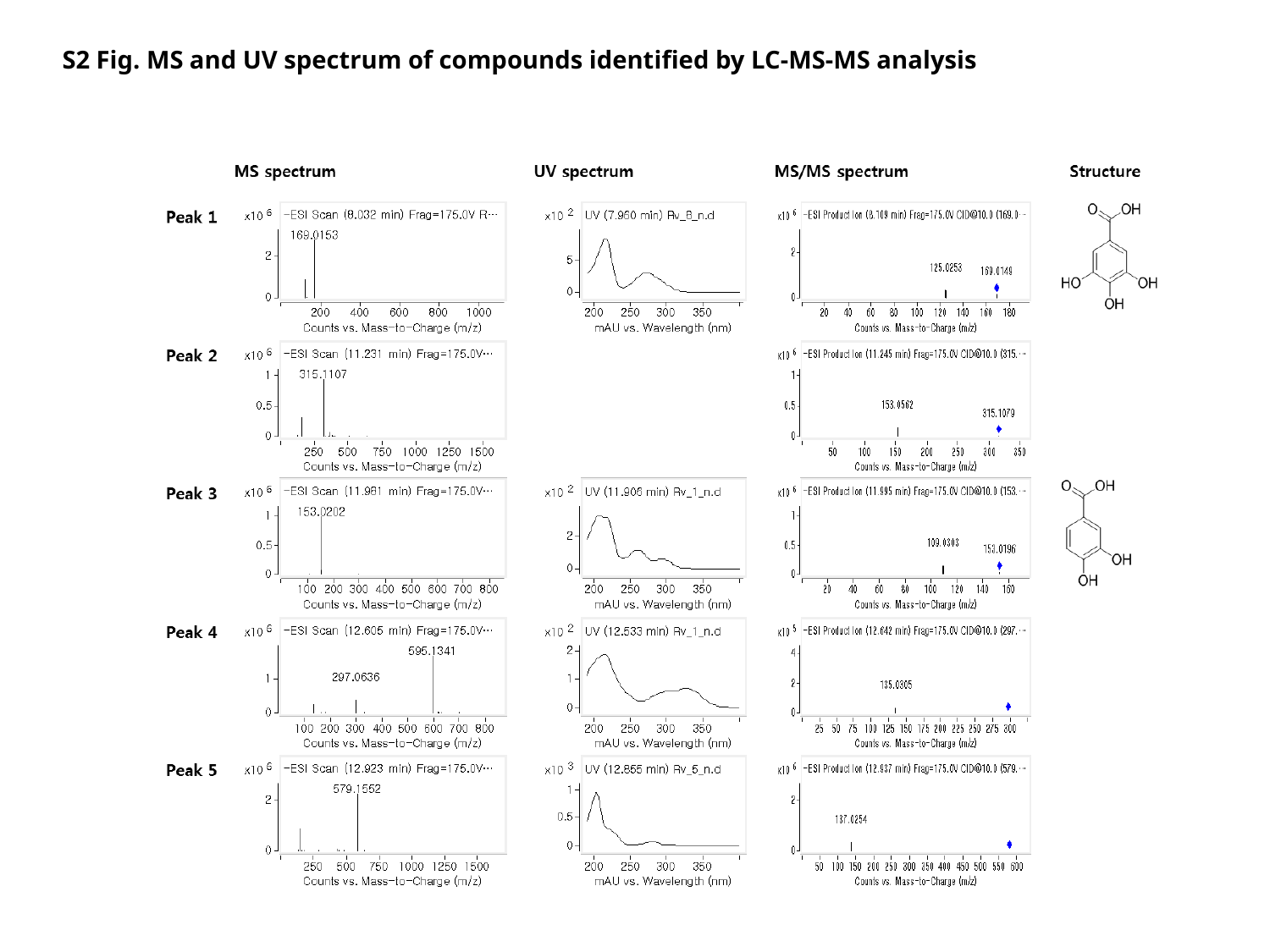

S2 Fig. MS and UV spectrum of compounds identified by LC-MS-MS analysis

## Slide 2
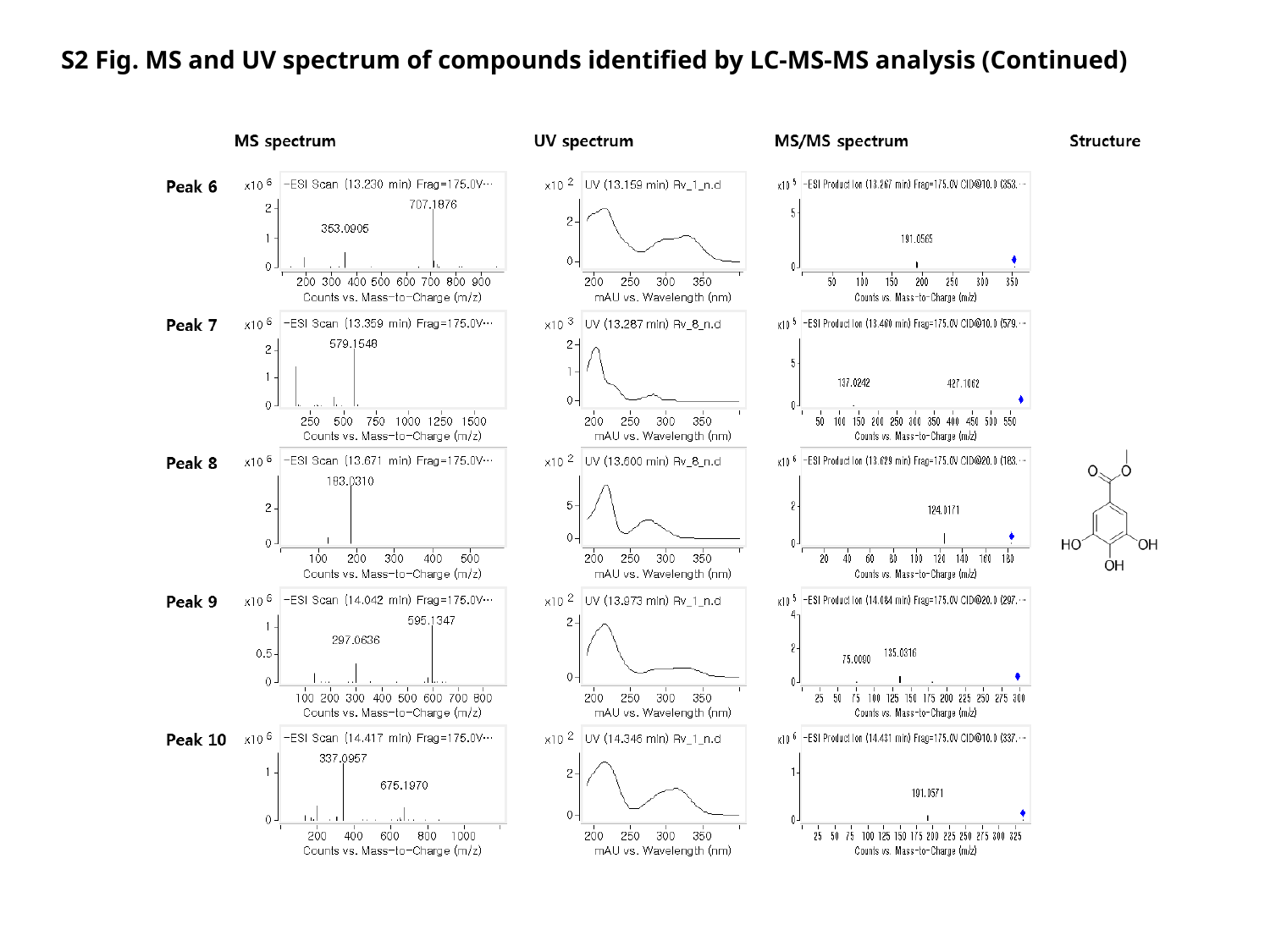

S2 Fig. MS and UV spectrum of compounds identified by LC-MS-MS analysis (Continued)

## Slide 3
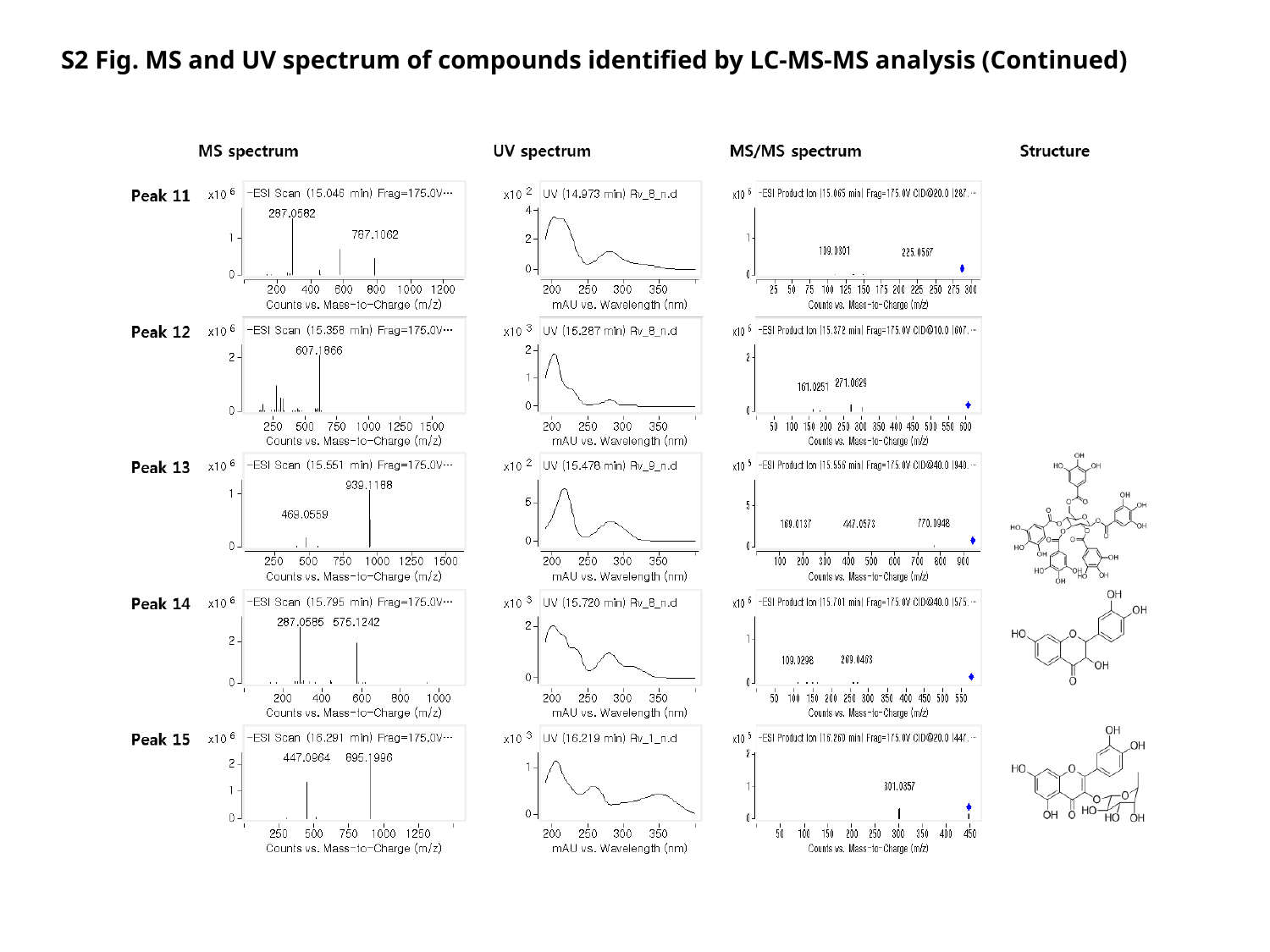

S2 Fig. MS and UV spectrum of compounds identified by LC-MS-MS analysis (Continued)

## Slide 4
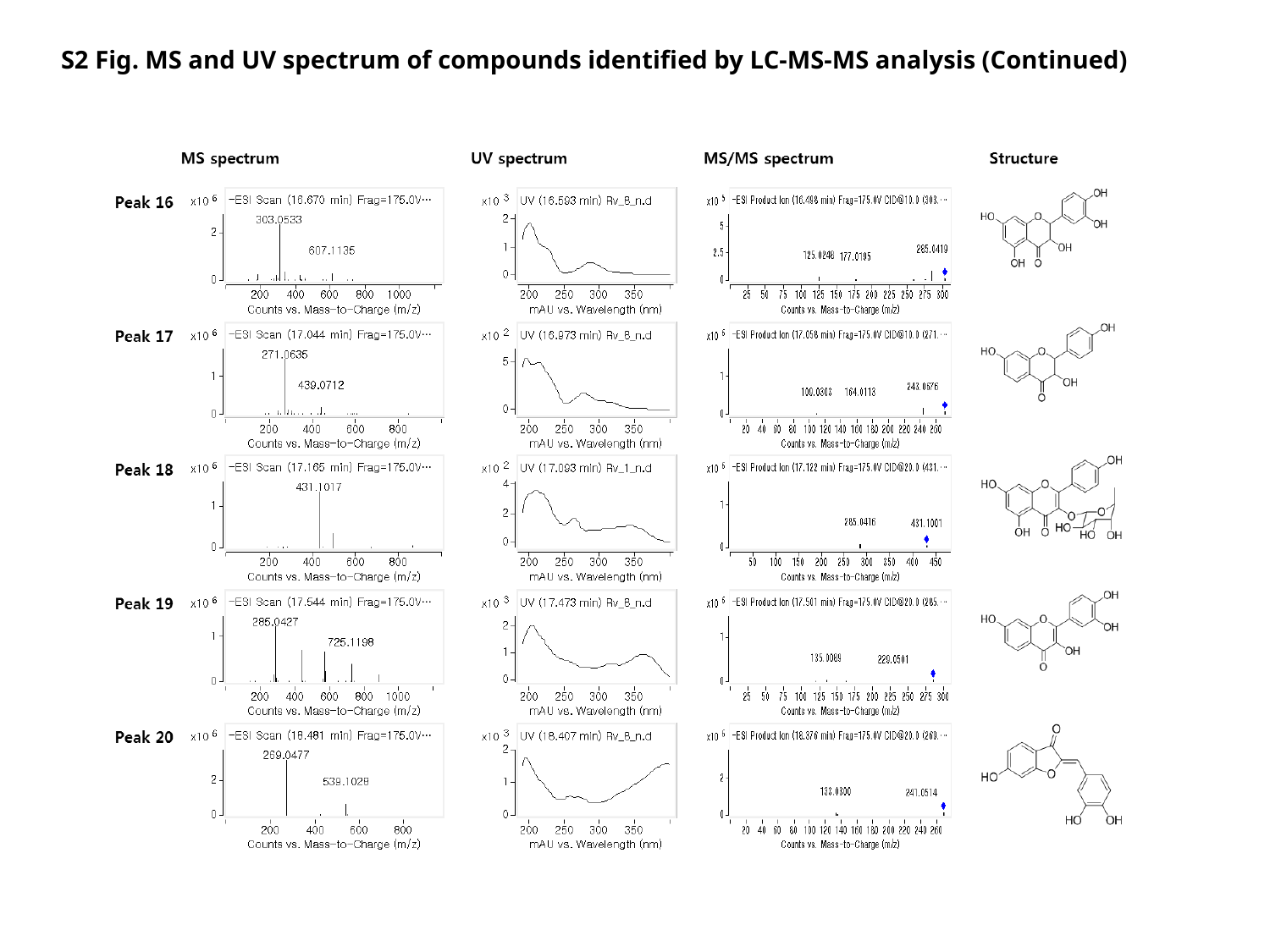

S2 Fig. MS and UV spectrum of compounds identified by LC-MS-MS analysis (Continued)

## Slide 5
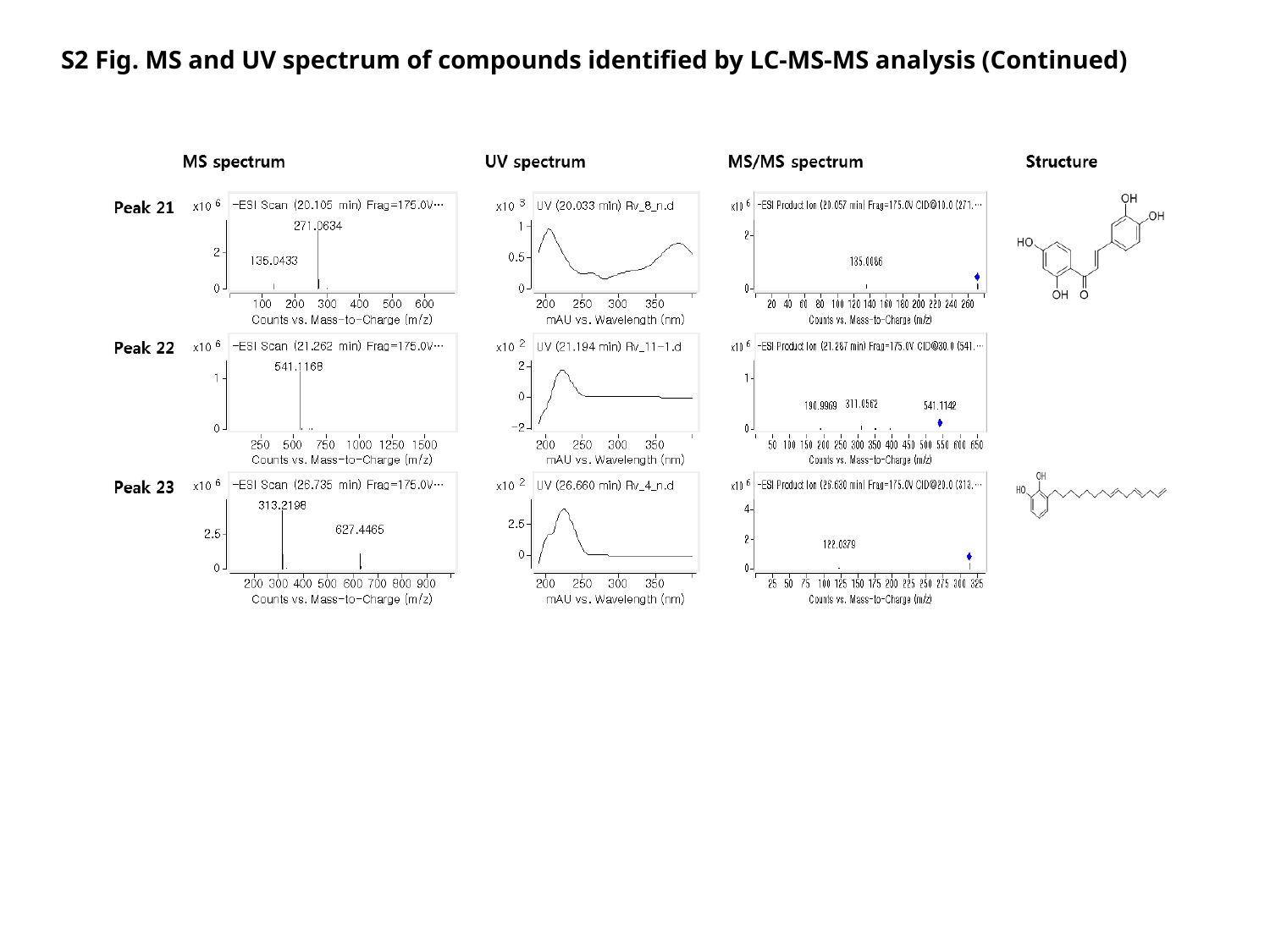

S2 Fig. MS and UV spectrum of compounds identified by LC-MS-MS analysis (Continued)
